# Patient, Family, and Peer Engagement in Nursing Care as an Effort to Improve the Functional Independence of Post-stroke Urinary Incontinence Patients: A Cross-Sectional Study

**DOI:** 10.7759/cureus.26649

**Published:** 2022-07-07

**Authors:** Heltty Heltty

**Affiliations:** 1 Medical-Surgical Nursing, Lembaga Layanan Pendidikan Tinggi Wilayah IX Sulawesi, Kendari, IDN

**Keywords:** post-stroke, urinary incontinence, functional independence, peer, family, patients, engagement

## Abstract

Introduction

The engagement of patients, family members, and peers is one approach that can be taken to improve the patient’s health status. This study aimed to investigate the relationship between patient, family, and peer involvement in nursing care to improve the functional independence of post-stroke urinary incontinence (UI) patients.

Methods

This cross-sectional descriptive design study was conducted in three hospitals in Kota Kendari, Sulawesi Tenggara, Indonesia. A total of 104 patients were selected using a simple random sampling method. Data were collected during the research period through a survey and observation. Data were analyzed using descriptive analysis and the Mann-Whitney test.

Results

There was a statistically significant difference in the motor subscale of the Functional Independence Measure (motor-FIM) domain (p<0.05). Based on the results of the analysis, there was a relationship between each motor-FIM domain in the engagement group.

Conclusions

The involvement of patients, families, and peers in patient care needs to be comprehensively improved in an effort to increase patient independence, which in turn can improve the quality of life of post-stroke urinary incontinence patients.

## Introduction

Post-stroke urinary incontinence (UI) is a sequela of acute stroke and is a strong predictor of high mortality, greater institutionalization, and increased disability [1]. International studies show that the average prevalence of UI was 8.2%-26.8% in 2016, of which 13%-38.7% occurred in women and 2.9%-9.9% in men. When compared with the elderly population, the prevalence of UI in the elderly reached an average of 29.4%, where 26.7%-36.3% of this number occurred in women and 6.4%-17% occurred in men [2].

UI is still a challenge for many stroke survivors and affects their quality of life [3]. Several studies have reported that post-stroke UI affects the quality of life [4,5]. The results of the identification of five studies of stroke patients with UI found that patients experienced a significant decrease in their quality of life and well-being, experienced a decrease in social participation, were significantly less independent in basic self-care, had a greater dependence on basic daily activities, had communication disorders, and had cognitive limitations [4]. Cross-sectional results at a month post-stroke (n=407) at Denmark Neurological Unit found that activity limitation was strongly associated with incontinence [4]. On average, post-stroke UI patients can only maintain 40% of their daily activities, while stroke patients without UI can maintain 70% of their daily activities [4].

UI is associated with physical weakness. UI risk increases as physical weakness increases [1]. Post-stroke UI patients experience dependence due to limitations or the physical weakness they experienced, so they are unable to carry out daily activities. Limitations in carrying out activities of daily living can prevent a person from using the toilet on time, therefore causing functional UI [6].

Behavioral intervention is recommended as the first line of therapy for UI management [7,8]. Behavioral treatments improve bladder control by changing the patient’s UI behavior, particularly urinary habits, and teaching skills to prevent urine leakage [9]. Behavioral interventions include bladder retraining, pelvic floor muscle training, and a rehabilitation approach [9]. Bladder retraining requires the patient to actively participate in treatment [10]. Individuals/patients who perform pelvic floor muscle exercises must have confidence and be motivated to perform the exercises regularly despite obstacles or difficulties in daily life [11]. For this reason, it is necessary to involve patients in their own care. However, post-stroke UI patients with physical weakness and psychosocial problems require help or support from family and peers.

Previous research has shown the effectiveness of treatment by involving the family in patient care [12]. Family members play a particularly important role when the patient is physically or cognitively unable to participate in self-care, and family members become surrogate decision-makers [13]. Family involvement in patient care is often recommended as a source of emotional, instrumental, informational, and affirmative support for people with chronic disease conditions [14]. The results of previous studies found that family played an important role in improving the quality of life of post-stroke UI patients, where there was an increase in the quality of life by 6.43 times at four weeks and 13 times at eight weeks, measuring the quality of life using the Incontinence Quality of Life (I-QOL) instrument [15].

In addition to family, attention from peers who also experienced the same disease can help reduce feelings of isolation and fear, where peer interaction can be done by sharing experiences and providing information about the health services they need [14]. The results of our previous qualitative research found that the presence of peers in the patient care process made the informants (patients) feel happy and not alone in dealing with their illness, making the informants became more eager to recover [16]. This study aimed to investigate the relationship between patient, family, and peer involvement in nursing care to improve the functional independence of post-stroke UI patients.

## Materials and methods

Design, sample, and setting

This cross-sectional descriptive design study was conducted in three hospitals in Kota Kendari, Sulawesi Tenggara, Indonesia (Regional Public Hospital Kota Kendari, Aliyah I Hospital, and Indonesian Army Hospital Dr. R. Ismoyo). Overall, 104 patients were selected using a simple random sampling method. The respondents who were eligible to participate in this study were stroke survivors aged 35-75 years and who underwent post-stroke from 48 hours to 8 weeks after the acute stroke phase, experienced urinary incontinence post-stroke, had a medically stable condition, are able to communicate verbally, and are not experiencing cognitive impairment that is known from the results of assessment using the Indonesian version of the Montreal Cognitive Assessment (MoCa-Ina) with a value of ≥25.

The researchers recruited eligible respondents in the rehabilitation unit and were assisted by research assistants (trained nurses) from each hospital where the study was conducted. The researchers conveyed the purpose of the research and explained the rights of the respondents, including the right to withdraw from the research. The researchers also said that the researchers maintain the confidentiality, anonymity, and security of the respondent’s personal data.

Data collection

Data collection was carried out from May to December 2021. Patient, family, and peer involvement was assessed through a survey. Patient, family, and peer involvement questionnaires were administered to each respondent. The researchers and research assistants help respondents explain each question item contained in the questionnaire. Completely filled out questionnaires were stored in a box provided at the rehabilitation unit. The respondent’s functional ability instrument was measured by a research assistant.

Ethical consideration

The research ethics committee at Regional Public Hospital Kota Kendari (IRB1429-1), Aliyah I Hospital (IRB256-01), and Indonesian Army Hospital Dr. R. Ismoyo (IRB02-1) approved this study. All respondents signed the informed consent form after receiving the research explanation. All respondents also received compensation in the form of educational information related to post-stroke UI.

Outcome measures

The quantitative data of patient demographics, patient, family, and peer engagement assessment, and functional independence were collected.

Demographics

The respondents provided data about their age, gender, marital status, and education. Clinical information included comorbidity, type of stroke, type of hemiparesis, type of UI, severity of UI, and severity of stroke using the National Institutes of Health Stroke Scale (NIHSS).

Patient, family, and peer engagement

The format of the assessment instrument was developed based on a literature study and expert consultation. Assessment items included patients and families being given health education related to their illness, patients and families being given clinical skills training, patients and families being involved in decision-making, patients and families being involved in patient self-care, families facilitating patients in carrying out health control to the hospital, peers sharing their experiences during treatment, and peers encouraging patients. This instrument uses a Likert scale with a rating score of 0-2 (not engaged, sometimes, and engaged), with a total score of 0-16; the higher the patient score, the higher the engagement of patients, families, and peers in patient care. All respondents’ assessments were collected, and then, the cutoff point was sought to determine the engagement and partial engagement groups.

Motor subscale of the Functional Independence Measure (motor-FIM)

The motor subscale of the Functional Independence Measure (motor-FIM) instrument is a basic indicator of patient disability. Motor-FIM is used to track the changes in the patient’s functional ability during rehabilitation care in the hospital. The motor subscale includes eating, grooming, bathing, dressing (upper body and lower body), toileting, bladder management, bowel management, transfers (bed/chair/wheelchair, toilet, bath/shower), walk/wheelchair, and stairs. The score of each motor-FIM assessment item is 1-7, with a total score of 13-91; the higher the score, the higher the patient’s level of independence. Each item was grouped into four domains: self-care, sphincter control, transfer, and locomotion. The motor-FIM has excellent internal consistency where the Cronbach’s alpha value is 0.88-0.91 and the validity value is 0.83-0.87 [17].

Data analysis

Data were analyzed using descriptive analysis and the Mann-Whitney test for the bivariate analysis. Descriptive analysis was conducted to describe the respondent’s demographic data and clinical information. A 95% confidence interval (CI) and a significant level of p<0.05 were used in this study. All analyses were done using SPSS Statistics version 25 (IBM Corp., Armonk, NY, USA).

## Results

The determination of the two groups was based on a cutoff design using the mean score. Values ​​above or equal to the mean score were designated as the engagement group, and values below the mean score were designated as the partial engagement group. Most of the respondents were female (58.7%) in both groups (the engagement group and the partial engagement group). The mean age in both groups was 54.69 years. Most of the respondents were married (53.3%), with the highest level of education being high school graduates (43.8%). All respondents were diagnosed with non-hemorrhagic stroke (100%). These respondents experienced more left-sided hemiparesis (59.6%) than right-sided hemiparesis. The average NIHSS score was 10.11 (moderate stroke). The most common comorbidities experienced by the respondents were hypertension (40.4%), and 43.3% had mixed incontinence (functional, stress incontinence, and urge incontinence). The majority of the respondents reported severe and very severe urinary incontinence (42.3% and 42.3%, respectively) symptoms (Table 1). The average age of the families involved in the two groups was the same (43.23 years). The most education level of the family in the engagement group was a bachelor’s degree (43.9%), while in the partial engagement group, it was high school graduates (57.4%). The families involved in patient care on average were spouses (42.9%), children (41.9%), parents (8.6%), and siblings (6.6%).

**Table 1:** Homogeneity Test of General Characteristics in the Engagement Group and the Partial Engagement Group DM: diabetes mellitus; M: mean; SD: standard deviation; UI: urinary incontinence

Characteristics and variables		Total (n (%) or M±SD)	Engagement	Partial engagement	p-value
Age (years)		54.69±11.72	48.07±10.74	62.72±6.861	0.000
Degree of the severity of stroke		10.11±2.19	8.96±1.92	11.49±1.64	0.437
Gender	Male	43 (41.3)	26 (45.6)	17 (36.2)	0.330
	Female	61 (58.7)	31 (54.4)	30 (63.8)	
Education level	Bachelor’s degree	32 (30.5)	28 (49.1)	4 (8.5)	0.000
	High school	46 (43.8)	22 (38.6)	24 (51.1)	
	Primary school	26 (24.8)	7 (12.3)	19 (40.4)	
Marital status	Married	56 (53.3)	37 (64.9)	19 (40.4)	0.013
	Not married (widow/widower/single)	48 (45.7)	20 (35.1)	28 (59.6)	
Types of hemiparesis	Left-sided hemiparesis	62 (59.6)	36 (63.2)	26 (55.3)	0.417
	Right-sided hemiparesis	42 (43.4)	21 (36.8)	21 (44.7)	
Comorbidities	Hypertension	42 (40.4)	30 (52.6)	12 (25.5)	0.016
	Hypertension and DM	36 (34.6)	17 (29.8)	19 (40.4)	
	Hypertension, DM, and hypercholesterolemia	26 (25)	10 (17.5)	16 (34)	
Types of UI	Functional	26 (25)	23 (40.4)	3 (6.4)	0.000
	Mixed (functional and stress)	33 (31.7)	24 (42.1)	9 (19.1)	
	Mixed (functional, stress, and urgency)	45 (43.3)	10 (17.5)	35 (74.5)	
Degree of the severity of UI	Moderate	16 (15.4)	15 (26.3)	1 (2.1)	0.000
	Severe	44 (42.3)	35 (61.4)	9 (19.1)	
	Very severe	44 (42.3)	7 (12.3)	37 (78.7)	

Analysis of the homogeneity test on both variables (motor-FIM and engagement) showed p<0.05. The test was continued with the Mann-Whitney test. The results of the Mann-Whitney analysis (Table 2) show that there was a statistically significant difference in the motor-FIM domain (p<0.05). Based on the results of the analysis, there was a relationship between each motor-FIM domain in the engagement group and the partial engagement group. However, we found that the achievement of average scores was higher in each of the motor-FIM domains (self-care, sphincter motor, transfer, and locomotion) in the engagement group.

**Table 2:** Achievement of Functional Independence in the Engagement Group and the Partial Engagement Group FIM: Functional Independence Measure; M: mean; SD: standard deviation; U: Mann-Whitney U; *: Mann-Whitney test

Domain motor-FIM	Engagement group (n=57)	Partial engagement group (n=47)		U	p-value*
	M±SD		M±SD		
Self-care	32.63±2.99		21.15±2.74	23.500	0.000
Sphincter motor	8.75±1.17		6.21±0.72	101.500	0.000
Transfer	12.98±1.33		9.15±0.72	30.500	0.000
Locomotion	8.72±0.99		6.30±0.83	136.000	0.000

## Discussion

The engagement of patients, family members, and peers is one approach that can be taken to improve the patient’s health status. Picker’s eight dimensions of patient-centered care provide a conceptual framework that emphasizes the involvement of family and friends in decision-making [18]. The results of this study show that the involvement of patients, family, and friends has a significant relationship to the patient’s functional independence (p<0.05). These results can be seen in the four motor-FIM domains showing higher scores in post-stroke UI patients who have family and friends involved in their care.

The results of the measurement of the self-care domain (feeding, grooming, bathing, dressing, and toileting) on the motor-FIM in this study showed an average level of self-care achievement of 5.43, which means that the average patient in the engagement group can carry out their self-care activities at the level under supervision. It suggests that the involvement of patients and families affects the attitudes and motivation of post-stroke patients to achieve independence [19]. Intentions toward independence will direct their attention toward managing self-care tasks during the rehabilitation period [19].

The results of the measurement of the motor sphincter domain (bladder and bowel management) on the motor-FIM in this study showed an average level of achievement of 4.38, which means that the average patient in the engagement group received minimal assistance in bladder and bowel management. Minimal assistance indicates that the patient needs little assistance in completing activities related to toileting. This is associated with increased recovery of the patient’s motor skills. Based on the respondent’s characteristic data, almost all patients experienced functional UI. In stroke patients, recovery of gross motor skills occurs spontaneously, but recovery of fine motor skills requires special and intensive training that involves certain muscles [20]. The recommended exercises to improve the patient’s urinary ability include pelvic floor muscle training and bladder retraining. It requires the active participation of the patient. A previous study indicated that pelvic floor muscle training and bladder retraining can improve the patient’s ability to manage their condition related to UI, therefore improving the quality of life related to urinary incontinence in post-stroke UI patients [15]. Improved quality of life can indicate increased patient independence.

The results of the transfer domain (transfers to and from bed/chair, toilet, and tub-shower) measurement on the motor-FIM in this study showed an average level of achievement of 4.33. It means that the average patient in the engagement group received minimal assistance in carrying out transfer movements. Transfer is a major ability to participate in activities of daily living and an important ability for patients to perform safely to increase their activity level [21]. The involvement of patients, family, and peers helps to achieve this ability. Likewise, the locomotion domain (walk/chair and stairs) measurement results on the motor-FIM in this study showed an average level of achievement of 4.36 (minimum assistance).

The achievement of the patient’s motor skills cannot be separated from the health education provided to the patient and his family. In this study, one of the engagement questions was the provision of health education. Providing health education to patients and family members is the first step in involving patients and families in nursing care. The knowledge of patients and families about post-stroke recovery significantly affects the patient’s rehabilitation outcomes [19]. In addition, respondents who participated in this study were respondents who could communicate verbally. It can improve the patient’s ability to receive and implement the health education that is taught.

The average family relationship most involved in patient care is spouse (42.9%) and children (41.9%). Spouses who are involved in patient care face new roles, although several studies reveal that changing partner roles are considered a burden both emotionally, mentally, and physically [22]. However, several studies have also shown that help and support from a spouse can motivate patients to carry out their daily tasks [23]. Apart from spouses, the majority of family members involved are children. This can be related to eastern cultural norms that uphold family values, teach children to take care of elderly parents, and as a basic moral obligation of adult children to care for sick and elderly parents [24].

Peer involvement in the form of support can help reduce feelings of isolation and fear by sharing experiences and providing information about needed health services [14]. Moderate to severe stroke patients who received social support showed a significantly improved functional status compared to those who received less support [25].

However, there are some limitations in this study, including the small sample size. Future research is needed to determine if these results are reproducible in different settings with a larger study population.

## Conclusions

The involvement of patients, families, and peers in patient care was significantly related to the functional independence of post-stroke UI patients compared to patients, family, and peers who were not or less involved in patient care. Functional independence in this study can be seen from the four domains in the measurement of the motor-FIM (self-care, sphincter motor, transfer, and locomotion), which on average show the level of independence at the minimum level of assistance. The involvement of patients, families, and peers in patient care needs to be comprehensively improved in an effort to increase patient independence, which in turn can improve the quality of life of post-stroke UI patients.
